# Upregulation of lncRNA SNHG1 is associated with metastasis and poor prognosis in cancers: A meta-analysis

**DOI:** 10.1097/MD.0000000000015196

**Published:** 2019-04-19

**Authors:** Jing Yu, Yunmeng Yan, Chunlan Hua, Liang Ming

**Affiliations:** a Department of Clinical Laboratory, The First Affiliated Hospital of Zhengzhou University; b Department of Clinical Laboratory, Key Clinical Laboratory of Henan Province, Zhengzhou, China.

**Keywords:** cancer, lncRNA small nucleolar RNA host gene 1, meta-analysis, metastasis, prognosis

## Abstract

**Background:**

Accumulating evidence suggested that the expression level of long noncoding RNA small nucleolar RNA host gene 1 (lncRNA SNHG1) was upregulated in various cancers, and high expression of SNHG1 was associated with metastasis and prognosis in patients with cancer.

The relationship between SNHG1 expression and metastasis or prognosis in malignant tumors was investigated in this meta-analysis.

**Methods:**

A systematic search was performed in PubMed, Web of Science, and Cochrane Library from inception until May 31, 2018. Hazard ratio (HR) or odds ratio (OR) with 95% confidence intervals (95% CIs) were calculated to demonstrate prognostic value of SNHG1 using Stata 12.0 software.

**Results:**

A total of 10 studies including 1129 patients were finally enrolled in the meta-analysis based on the inclusion and exclusion criteria. Increased SNHG1 expression was significantly associated with lymph node metastasis (OR = 3.28, 95% CI = 2.02–5.33) and advanced TNM stage (OR = 0.26, 95% CI = 0.16–0.43). Moreover, high expression of SNHG1 could predict poor overall survival (HR = 2.32, 95% CI = 1.90–2.83), event-free survival (HR = 1.58, 95% CI = 1.06–2.35), recurrence-free survival (HR = 2.15, 95% CI = 1.23–3.77), progression-free survival (HR = 2.75, 95% CI = 1.70–4.46), and disease-free survival (HR = 1.93, 95% CI = 1.10–3.40) in patients with cancer.

**Conclusion:**

The present meta-analysis demonstrated that upregulation of lncRNA SNHG1 might serve as a useful prognostic biomarker in various cancers.

## 1 Introduction

With the increasing of morbidity and mortality in each year, cancer has been a major public health problem worldwide.^[1]^ Although comprehensive treatment strategies for patients with cancer have been developed, such as surgery, chemoradiotherapy, and targeted therapy,^[2]^ the clinical outcome is still very poor in many cancers, which may be due to lacking effective and prompt diagnostic methods. Therefore, it is urgently needed to identify specific biomarkers for early diagnosis and prognosis of patients with cancer.

Long noncoding RNAs (lncRNAs) are a class of transcribed RNA molecules more than 200 nucleotides in length without protein-coding capacity.^[3]^ Accumulating evidence revealed that dysregulated expression of lncRNAs was involved in various biologic progress in cancers, such as cell proliferation, cell apoptosis, cell invasion, cell differentiation, and carcinogenesis.^[4–6]^ Moreover, aberrant lncRNA expression was found to lead to metastasis and cancer progression.^[7–9]^ The lncRNAs such as urothelial cancer associated 1 (UCA1),^[10]^ metastasis-associated lung adenocarcinoma transcript 1 (MALAT1),^[11]^ H19,^[12]^ and PVT1^[13]^ were found to predict lymph node metastasis (LNM) and a poor prognosis in human cancers. Recently, the lncRNA small nucleolar RNA host gene 1 (SNHG1) was found to be high-expressed and functioned as an oncogene in various cancers, such as nonsmall-cell lung cancer,^[14]^ gastric cancer,^[15]^ and colorectal cancer.^[16]^ Previous studies have revealed that upregulated SNHG1 expression predicted poor prognosis for some cancers.^[17–19]^ Meanwhile, SNHG1 express level was correlated with LNM, distant metastasis (DM) and TNM stage in various cancers.^[20,21]^ To date, no meta-analysis has been performed to examine the relationship between SNHG1 and the relevant clinical outcomes. Therefore, we carried out this meta-analysis, including 10 studies and 1129 patients, to explore the relationships between SNHG1 expression and the clinical pathologic parameters and prognosis in human cancers.

## 2 Materials and methods

A systematic search of the electronic databases including PubMed, Cochrane Library, and Web of Science was performed to obtain relevant articles for the meta-analysis. Studies were selected using the following key words: “long noncoding RNA SNHG1,” “lncRNA SNHG1,” “small nucleolar RNA host gene 1,” “tumor,” “cancer,” and “carcinoma.” Other relevant studies were also obtained by manually screening the references list.

### 2.1 Inclusion criterion

The inclusion standards are as follows: studies investigating the clinical role of SNHG1 in cancers; patients were divided into 2 groups (high-expression group and low-expression group) according to the expression levels of SNHG1; associations of SNHG1 expressions with clinicopathologic features, overall survival (OS) were described. The excluded standards are as follows: duplicate publications; studies with insufficient or unavailable data; letters, reviews, case reports, and expert opinions.

### 2.2 Data extraction

Two investigators performed the data extraction independently. The following information was extracted: 1st author, publication year, country, cancer type, detection method of SNHG1 expression, number of patients, TNM stage, follow-up period, outcome, hazard ratio (HR) estimate, and cut-off values. Any discrepancies between 2 investigators were resolved by discussion until reaching a consensus.

### 2.3 Statistical methods

We used Stata SE12.0 (Stata, College Station, TX) to estimate HRs for OS, MFS or recurrence-free survival (RFS), and odd ratios (ORs) for clinicopathologic parameters. The heterogeneity among the studies was evaluated by the Chi-squared value and the *I*^2^ value. If *I*^2^ ≤ 50% or *P* > .05, a fixed-effects model was used for analysis. If not (*I*^2^ > 50% or *P* ≤ .05), a random-effects model was used. The Stata SE12.0 was used to evaluate the sensitivity and publication bias of the studies. Publication bias was evaluated by Begg and Egger tests. *P*-values <.05 were considered statistically significant.

## 3 Results

### 3.1 Study selection

**Figure 1:**
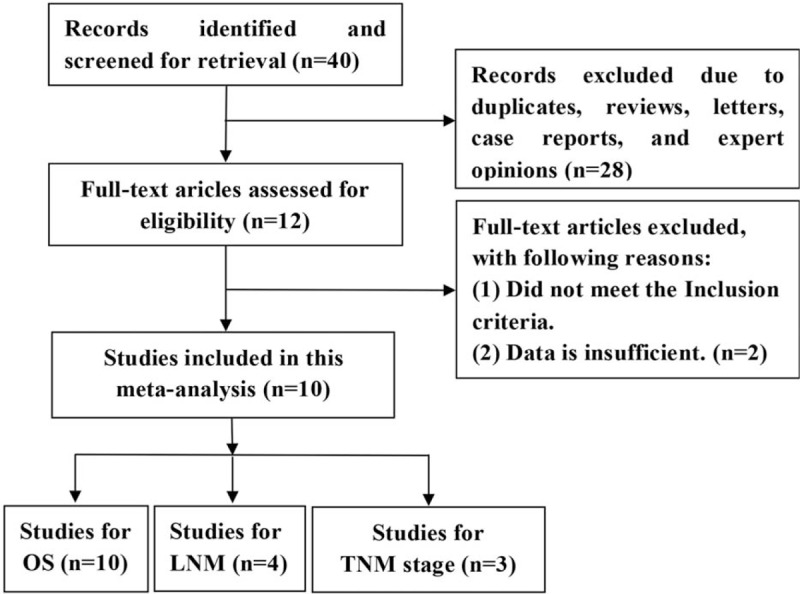
The flow chart of study selection procedure in the meta-analysis.

As shown in the flow diagram (Fig. 1), 40 published articles are obtained through electronic searches. After screening the title and abstract carefully, 28 articles are excluded. Then, 2 papers are excluded due to the lack of data availability. Finally, a total of 10 studies are included in the meta-analysis.^[15,17–25]^ Total 1129 participants are included in these studies. The main characteristics of the included studies are shown in Table 1.

**Table 1:**
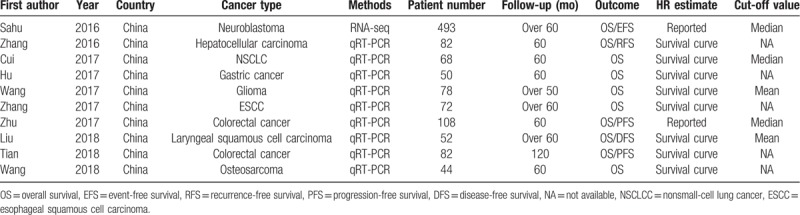
Characteristics of the studies included in the meta-analysis.

### 3.2 Association between SNHG1 expression levels and OS

**Figure 2:**
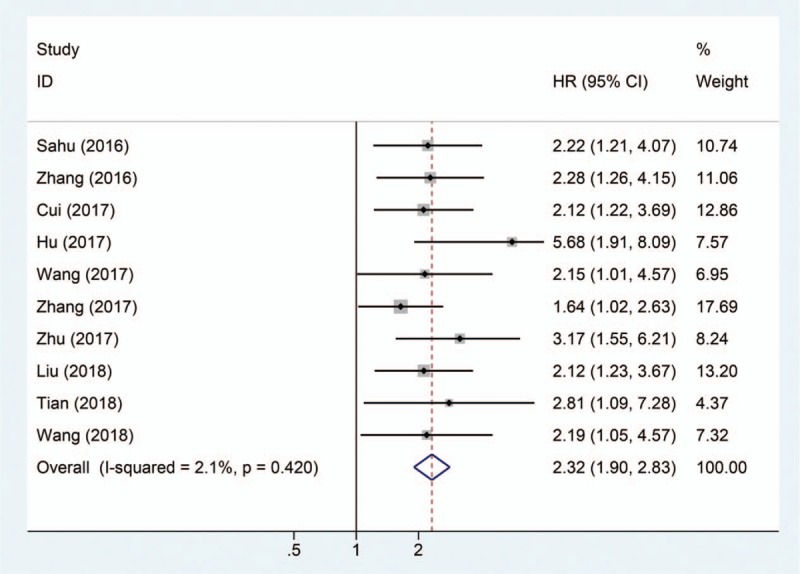
Forest plot shows that elevated lncRNA SNHG1 expression is associated with poor overall survival.

The association between SNHG1 and OS is shown in Figure 2. Ten studies, including 1129 patients, were included in this meta-analysis of OS. Since there is no significant heterogeneity (*P* for the heterogeneity = .420, *I*^2^ = 2.1%), a fixed-effects model is applied to calculate the pooled HRs and the respective 95% confidence interval (CI). The HR, expressed as the high SNHG1 expression group versus the low SNHG1 expression group, is 2.32 (95% CI = 1.90–2.83, *P* < .001) (Fig. 2). The result reveals that elevated lncRNA SNHG1 expression is associated with poor OS.

**Figure 3:**
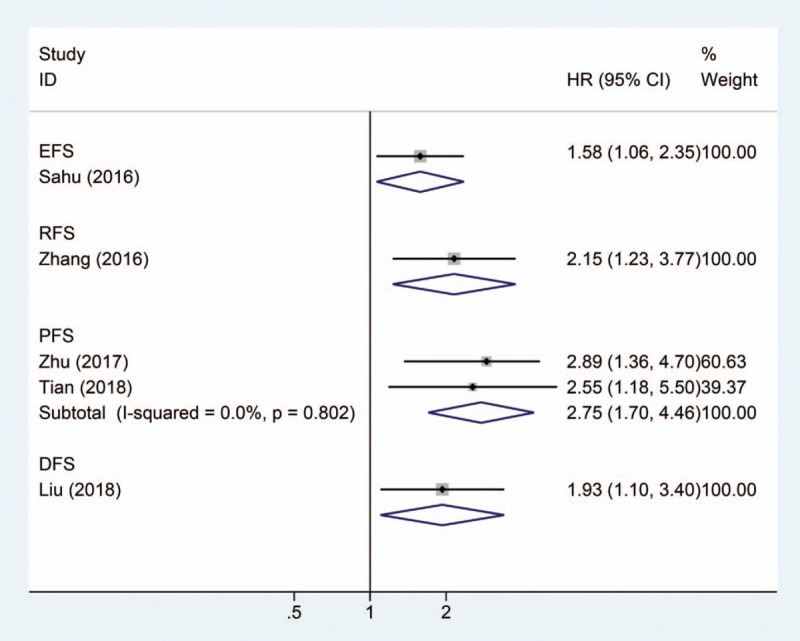
Forest plot shows that high SNHG1 expression is associated with poor free survivals.

The association between SNHG1 expression and event-free survival (EFS), RFS, progression-free survival (PFS), and disease-free survival (DFS) are reported in Figure 3. Analysis shows an HR of 1.58 with 95% CI = 1.06–2.35 for EFS, an HR of 2.15 with 95% CI = 1.23–3.77 for RFS, an HR of 2.75 with 95% CI = 1.70–4.46 for PFS, and an HR of 1.93 with 95% CI = 1.10–3.40 for DFS (Fig. 3), which indicates a significantly negative association between the expression levels of SNHG1 and EFS, RFS, PFS, or RFS.

### 3.3 Association between SNHG1 expression levels and LNM

**Figure 4:**
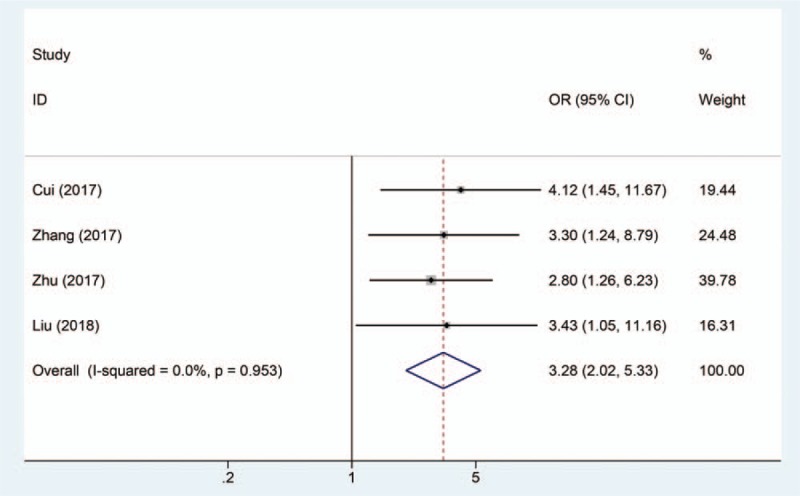
Forest plot shows that high SNHG1 expression is more prone to developing lymph node metastasis.

Four studies, including 300 patients, were analyzed for the association between SNHG1 expression and LNM incidence. A fixed-effects model was used due to no significant heterogeneity (*P* for the heterogeneity = .953, *I*^2^ = 0%). Our data demonstrated that high SNHG1 expression is more prone to developing LNM with a pooled OR of 3.28 (95% CI = 2.02–5.33, *P* < .001; Fig. 4).

### 3.4 Association between SNHG1 expression levels and TNM stage

**Figure 5:**
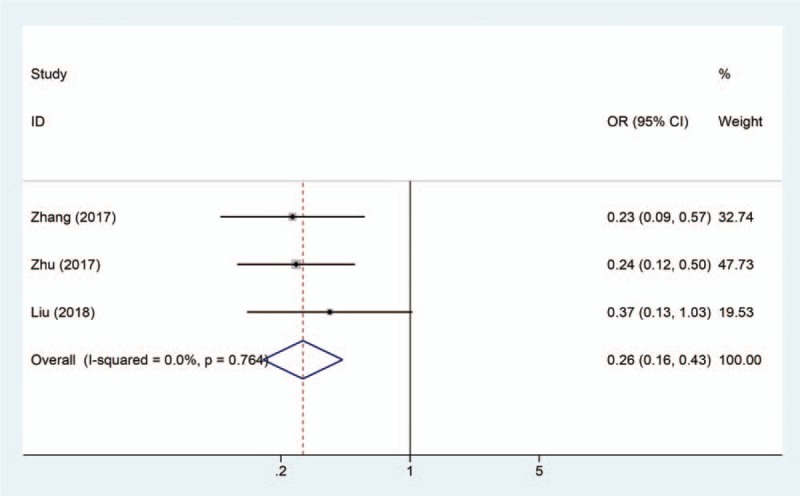
Forest plot shows that high SNHG1 expression is positively associated with advanced TNM stage.

Three studies including 232 patients reported the tumor stage based on different SNHG1 expression levels. The result displays a pooled OR = 0.26 (95% CI = 0.16–0.43, *P* < .001) in a fixed-effects model (*I*^2^ = 0%, *P* = .764) (Fig. 5). The result demonstrates that upregulated SNHG1 is positively related to advanced TNM stage (*P* < .001).

### 3.5 Association between SNHG1 expression levels and DM

**Figure 6:**
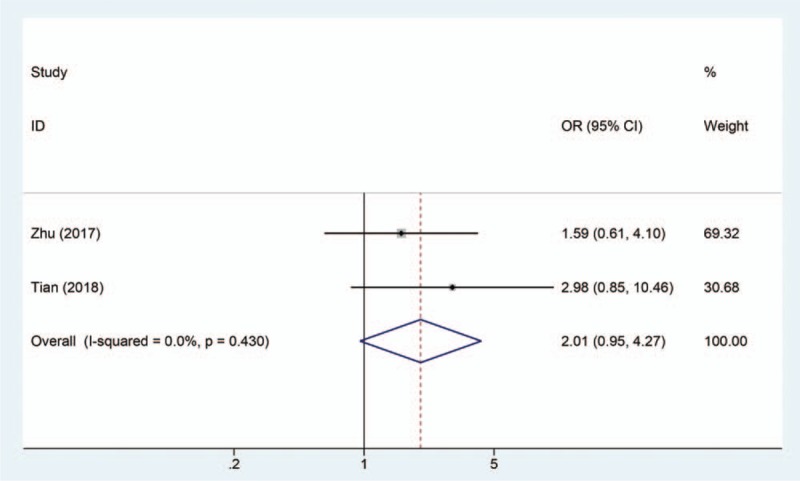
Forest plot that increased SNHG1 expression is more prone to developing distant metastasis.

Two studies including 190 patients reported the DM based on different SNHG1 expression levels. The result displays a pooled OR = 2.01 (95% CI = 0.95–4.27, *P* = .067) in a fixed-effects model (*I*^2^ = 0%, *P* = .430) (Fig. 6). The result demonstrates that increased SNHG1 expression is more prone to developing DM.

### 3.6 Sensitivity analysis

**Figure 7:**
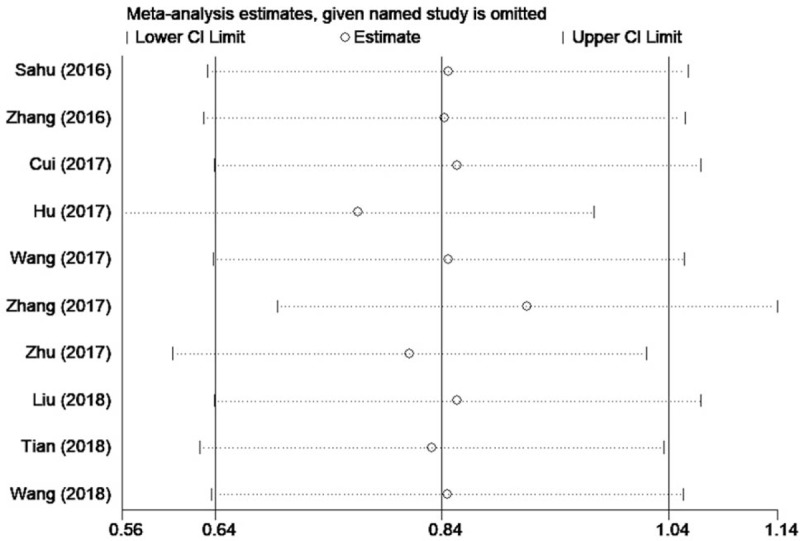
Sensitivity analysis for small nucleolar RNA host gene 1 expression with overall survival.

To determine whether the individual study exerted influence on the overall results of OS, the sensitivity analysis was performed. Our data suggest that removing any of the included studies has no significant influence on the results (Fig. 7), which demonstrate that our results are considerably reasonable and reliable.

### 3.7 Publication bias

**Figure 8:**
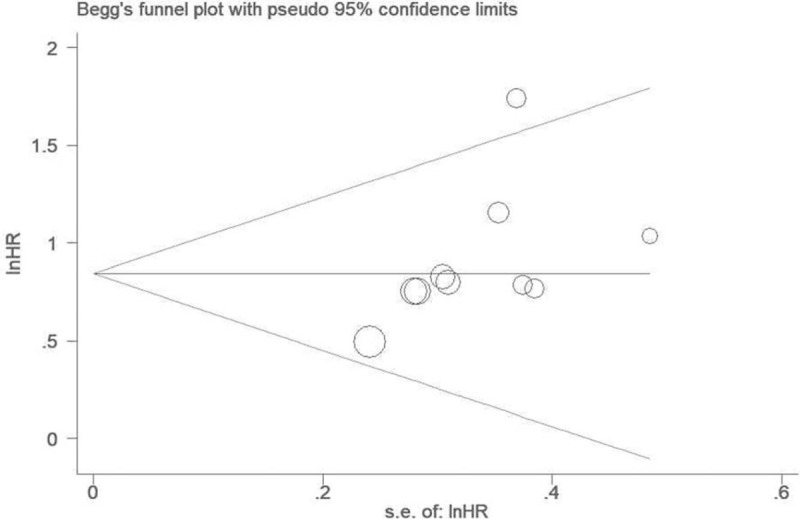
Funnel plot analysis shows that there is no publication bias for overall survival.

The publication bias was evaluated by Begg test and Egger test. The shape of the funnel plot is almost symmetrical for OS (*P* = .107) by Begg test (Fig. 8), which indicates that there is no obvious publication bias for OS. Moreover, the result of Egger test also shows that there is no obvious publication bias for OS (*P* = .07).

## 4 Discussion

Accumulating evidence revealed that abnormal expression of lncRNAs acts as oncogenes or tumor suppressors in various cancers.^[26–28]^ LncRNA has been demonstrated to function as molecular scaffolds, sponges or coactivators by interaction with DNA, RNA, or proteins. Many lncRNAs play a vital role in the progression of tumors, with involvement in tumor proliferation, invasion, and metastasis.^[29]^ Therefore, identification of tumor-related lncRNAs is important for understanding the function in tumorigenesis and providing promising therapeutic targets for patients with cancer.

Recently, many literatures suggested that SNHG1 was upregulated in various malignant tumors, such as gastric cancer, renal cell carcinoma, and nasopharyngeal carcinoma.^[15,30,31]^ SNHG1 was reported to be involved in cancer cell proliferation, apoptosis, invasion, and epithelial-mesenchymal transition. Hu et al found that SNHG1 was a negative prognostic factor and exhibited oncogenic activity in gastric cancer.^[15]^ Another study revealed that upregulation of SNHG1 was highly expressed in cervical cancer, which led to promotion of proliferation, migration, and invasion.^[32]^ Moreover, elevated SNHG1 expression promoted esophageal carcinoma cell proliferation and suppressed its apoptosis through elevating protooncogene CST3 expression by sponging miR-338.^[33]^ All these studies demonstrated that SNHG1 functioned as an oncogene in various cancers, which may be a promising diagnostic marker for patients with cancer.

Previously, SNHG1 was reported to be associated with clinical parameters and prognosis of patients with cancer and may be a potential diagnosis biomarker in several cancers.^[20,21]^ In this study, a meta-analysis was 1st conducted to investigate the correlation between SNHG1 expression level and clinicopathologic characteristics, and to evaluate the role of SNHG1 as a prognosis marker for patients with cancer. Ten studies including 1129 patients were pooled in this study, and the results indicated that lncRNA SNHG1 upregulation was significantly correlated with poor prognosis (HR = 2.32, 95% CI = 1.90–2.83, *P* < .0001). Moreover, there was negative correlation between SNHG1 levels and EFS (HR = 1.58, 95% CI = 1.06–2.35) in neuroblastoma, RFS (HR = 2.15, 95% CI = 1.23–3.77) in hepatocellular carcinoma, PFS (HR = 2.75, 95% CI = 1.70–4.46) in colorectal cancer, and DFS (HR = 1.93, 95% CI = 1.10–3.40) in laryngeal squamous cell carcinoma. Taken together, SNHG1 could serve as biomarker for the prognosis of patients with cancer. In this meta-analysis, the association betweenSNHG1 levels and clinicopathologic characteristics was further evaluated. We found that elevated SNHG1 level was more prone to lead to LNM (OR = 3.28, 95% CI = 2.02–5.33, *P* < .001), advanced TNM stage (OR = 0.26, 95% CI = 0.16–0.43, *P* < .001), and DM (OR = 2.01, 95% CI = 0.95–4.27, *P* = .067). Although elevated SNHG1 expression was associated with high DM, the correlation was not significant. Therefore, more studies were required to confirm the relationship between SNHG1 levels and DM.

Several limitations should be taken into consideration when interpreting the findings. First, the number of involved studies and patients was limited, especially for clinical parameters. Second, included studies were from China, which made that our data not represent globally. Third, the survival analyses in the studies did not provide specific HRs and 95% CI, and so, we calculated them by available software, which may have introduced errors. Therefore, these factors should be taken into account to conclude a true effect. Due to these limitations, the results presented by the present study should be interpreted with caution.

In summary, high SNHG1 expression in multiple cancers is significantly correlated with poor OS, LNM, advanced TNM stage and DM. Therefore, lncRNA SNHG1 expression may serve as a promising biomarker for predicting prognosis in patients with cancer.

## Author contributions

**Data curation:** Jing Yu, Chunlan Hua.

**Formal analysis:** Chunlan Hua.

**Investigation:** Jing Yu.

**Methodology:** Chunlan Hua.

**Supervision:** Yunmeng Yan, Liang Ming.

**Writing - Original Draft:** Jing Yu.

**Writing - Review & Editing:** Yunmeng Yan, Liang Ming.
